# Distinct evolutionary mechanisms for genomic imbalances in high-risk and low-risk neuroblastomas

**DOI:** 10.1186/1477-3163-6-15

**Published:** 2007-09-26

**Authors:** David Gisselsson, Gisela Lundberg, Ingrid Øra, Mattias Höglund

**Affiliations:** 1 Department of Clinical Genetics, Lund University Hospital, SE 221 85 Lund, Sweden; 2 Department of Pathology, Lund University Hospital, SE 221 85 Lund, Sweden; 3 Department of Paediatric Haematology and Oncology, Lund University Hospital, SE 221 85 Lund, Sweden

## Abstract

**Background:**

Neuroblastoma (NB) is the most common extracranial solid tumour of childhood. Several genomic imbalances correlate to prognosis in NB, with structural rearrangements, including gene amplification, in a near-diploid setting typically signifying high-risk tumours and numerical changes in a near-triploid setting signifying low-risk tumours. Little is known about the temporal sequence in which these imbalances occur during the carcinogenic process.

**Methods:**

We have reconstructed the appearance of cytogenetic imbalances in 270 NBs by first grouping tumours and imbalances through principal component analysis and then using the number of imbalances in each tumour as an indicator of evolutionary progression.

**Results:**

Tumours clustered in four sub-groups, dominated respectively by (1) gene amplification in double minute chromosomes and few other aberrations, (2) gene amplification and loss of 1p sequences, (3) loss of 1p and other structural aberrations including gain of 17q, and (4) whole-chromosome gains and losses. Temporal analysis showed that the structural changes in groups 1–3 were acquired in a step-wise fashion, with loss of 1p sequences and the emergence of double minute chromosomes as the earliest cytogenetic events. In contrast, the gains and losses of whole chromosomes in group 4 occurred through multiple simultaneous events leading to a near-triploid chromosome number.

**Conclusion:**

The finding of different temporal patterns for the acquisition of genomic imbalances in high-risk and low-risk NBs lends strong support to the hypothesis that these tumours are biologically diverse entities, evolving through distinct genetic mechanisms.

## Background

Neuroblastoma (NB) originates from the postganglionic cells of the sympathetic nervous system and is the most common extracranial solid tumour of childhood. Four of five children with NB are diagnosed before 4 years of age and it is the most common tumour in infancy, becoming less frequent in each succeeding year. In spite of vast knowledge about the biology of NB, the long-term survival is still less than 30% for children with high-risk tumours [1]. It is a well-known fact that acquired chromosome changes in neoplastic cells contribute to tumour development. This is well illustrated by NB, where several genomic imbalances have been shown to correlate to prognosis. Amplification of the *MYCN *oncogene in double minute chromosomes (dmin) or homogeneously staining regions (hsr), loss of heterozygosity for the short arms of chromosomes 1 (1p) and 3 (3p) and the long arm of chromosome 11 (11q), and gain of sequences from 17q have all been shown to predict a poor prognosis [2-5]. On the other hand, tumours with hyperdiploid or near-triploid karyotypes dominated by whole-chromosome gains and losses have a considerably better prognosis and may even undergo spontaneous regression [3,6,7]. The former pattern of imbalances is generally more common in patients above 12–18 months of age, whereas the latter one is more common in younger children.

Despite the importance of genomic imbalances for the clinical outcome of NB, little is yet known about the processes through which such imbalances are acquired. We have previously established a method for statistically assessing the relative time of occurrence (TO) of cytogenetic aberrations in tumours by determining the degree of cytogenetic complexity at which each aberration typically occurs [8-12]. The possibility to screen for genomic imbalances in NB at a high resolution level has improved dramatically over the last few years, with the introduction of array-based whole-genome screening techniques [3,13-15]. However, with some notable exceptions [15], studies of NB by such high-resolution screening methods have so far only included a relatively small number of cases. In contrast, several hundred NBs have been analysed by chromosome banding methodology and curated karyotypes of published cases are available in a public database [16]. In order to maximise the number of cases used for temporal reconstruction, we therefore chose to analyse data from this latter source, first by grouping imbalances frequently occurring together, and then by establishing their relative order within each group. We finally correlated these abnormalities to basic clinical parameters. Our data indicate that near-diploid, highly aggressive, tumours and near-triploid, less aggressive, tumours acquire their respective chromosome aberrations through different temporal processes: While the near-diploid tumours accumulate clonal abnormalities through sequential genomic rearrangements, the second group of tumours obtains their near-triploid status by multiple simultaneous events.

## Methods

### General strategy

Karyotypes from 270 NB patients available in the Mitelman Database of Chromosome Aberrations in Cancer were downloaded as text files. ISCN karyotypes [17] were converted into binary data sets denoting the status of each chromosome band as 1 if gained, -1 if lost, and 0 if neither gained nor lost. [18]. Whole genome profiles of all chromosomes were then created and chromosome segments gained or lost in >5% of the tumours were selected for further analysis (Table 1). In the event that one or more segments of a chromosome deviated >5% units from the whole-chromosome profile, these segments were scored separately from the whole-chromosome imbalances. Each karyotype was then assessed for the presence or absence of the selected imbalances. The number of imbalances per tumour (NIPT) was calculated and cases with at least one of the chosen imbalances were selected for further analysis, resulting in 259 cases. Also, if available, the age of the patient as well as tumour stage according to the International Neuroblastoma Staging System [19] was retrieved from the original publication of each case. Age and stage were combined into a simplified clinical risk stratification as follows: patients with stage 1-, 2- and 4s-disease were assigned low risk irrespective of age, whereas patients with stage 3-disease irrespective of age and stage 4-disease <1 years of age were assigned intermediate risk. Stage 4 cases >1 years of age were classified as high risk.

**Table 1:** Relative frequency and age/stage/risk correlates of cytogenetic abnormalities

Karyotype features	Frequencies (%)	Overepresented group (P value)		
*Ploidy level*		*Age*	*Stage*	*Risk*
2n	59	-	-	-
3n	23	<1 ys (6 × 10^-4^)	1–2 (4 × 10^-5^)	L (1 × 10^-4^)
4n	15	-	-	-
*Whole chromosome gains*				
+17	15	<1 ys (3 × 10^-4^)	1–2 (4 × 10^-5^)	L (7 × 10^-6^)
+7	13	-	-	-
+18	11	-	-	-
+12	10	<1 ys (3 × 10^-3^)	1–2 (2 × 10^-4^)	L (2 × 10^-4^)
+20	9	-	-	-
+13	9	-	-	-
+9	8	<1 ys (2 × 10^-4^)	1–2 (2 × 10^-8^)	L (3 × 10^-7^)
+6	7	-	-	-
+8	7	-	-	-
+21	6	-	-	-
+5	6	-	-	-
+11	5	-	-	-
+2	5	-	-	-
+1	5	-	-	-
*Whole chromosome losses*				
-X	21	-	-	-
-11	14	-	-	-
-10	14	-	-	-
-15	14	-	-	-
-19	14	-	-	-
-4	14	-	1–2 (5 × 10^-4^)	L (5 × 10^-4^)
-3	12	-	-	-
-9	11	-	-	-
-14	11	-	-	-
-17	11	-	-	-
-22	11	-	-	-
-21	11	-	-	-
-13	10	-	-	-
-16	10	-	-	-
-8	9	-	-	-
-6	9	-	-	-
-20	9	-	-	-
-5	8	-	-	-
-2	6	-	-	-
-18	6	-	-	-
-12	5	-	-	-
*Partial chromosome gains*				
+1q (1p31q44)	15	-	-	-
+17q (17q12q25)	13	-	-	-
*Partial chromosome losses*				
1p- (1p36p11)	48	-	-	H (4 × 10^-3^)
11q- (11q23q25)	8	-	-	-
3p- (3p26p12)	8	-	-	-
6q- (6q23q27)	6	-	-	-
*Gene amplifications*				
dmin	20	1–3 (8 × 10^-5^)	-	H (6 × 10^-3^)
hsr	6	-	-	-

Over-representation tested by Chi-Square test with significance level set at P < 0.01. Abbreviations: ys, years; L, low risk; H, high risk; -, not significantly over-represented in any subgroup.

### Statistical analyses

To search for possible patterns of correlations between the imbalances, principal component analysis (PCA) was performed using the Statistica software package (Statsoft, Tulsa, OK). PCA is a standard multivariate method frequently used to search for underlying structures in data sets [18]. In short, principal components are linear combinations of the original variables, orthogonal, and ordered with respect to their variance so that the first principal component has the largest variance. To analyse imbalances, these were used as variables and the individual tumours as the observations; this will group imbalances frequently seen in the same tumours. Tumour cases were analysed by using the tumours as variables and the imbalances as cases. This will group tumours with similar karyotypic profiles. The TO was determined essentially as described [8]. Briefly, all tumours with a given imbalance were selected and the distributions of NIPT plotted. The modes of the NIPT distributions were then used as values for TO. To obtain a better estimate of the TO, the selected distributions were re-sampled with replacement (bootstrapped) 1,000 times and the TO scored after each re-sampling [20]. The mean of the bootstrapped TO values was then used as the TO for the given imbalance and the bootstrapped 25th and 75th percentiles calculated. For the bootstrap estimates, re-sampling software from Resampling Stats (Arlington, VA) was used.

## Results

### Basic statistics

Of the 270 cases, 59% were near-diploid, 23% were near-triploid, and 15% were near-tetraploid. The remaining 3% either had ploidies defined as intervals spanning different ploidy levels or had multiple clones at different levels. In total, 14 whole-chromosome gains, 21 whole-chromosome losses, two segmental chromosome gains, and four segmental chromosome losses were found in >5% of the tumours (Table 1). Double minute chromosomes (dmin) and homogeneously staining regions (hsr) were also present in >5% of the karyotypes. One or more of these abnormalities were present in 259 (93%) of the total number of tumours.

### Principal component analysis of imbalances

**Figure 1:**
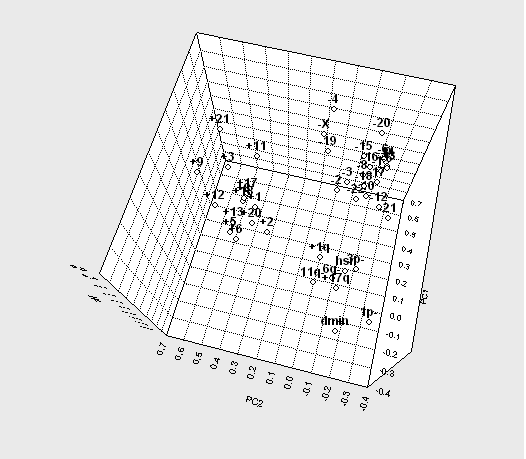
Principal component analysis of recurrent imbalances in neuroblastoma. A three-dimensional representation based on the three first principal components is shown. Three major clusters of imbalances are seen; one composed of all structural aberrations, one containing all whole chromosome gains, and one with all whole chromosome losses. PC1, PC2, PC3 are principal components 1, 2, and 3, respectively.

To assess whether some of the selected abnormalities preferentially occurred together with one or more other abnormalities, we performed PCA of the karyotypic data. This revealed three distinct clusters of aberrations, one containing all the whole-chromosome gains, a second containing all whole-chromosome losses, and a third containing all imbalances resulting from structural rearrangements, *i.e. *segmental gains, segmental losses, hsr and dmin (Figure 1). Although the distance between two aberrations in the PCA diagram is ultimately a representation of the correlation between the frequencies of these two abnormalities in any sub-set of tumours, the relationships between the abnormalities were often complex. For example, 1p- and dmin were located outside the main cluster of structural rearrangements and the presence of one of these changes correlated only weakly with the other (r = +0.13; P < 0.01). Of the total 57 cases with dmin, 35 showed 1p- (61%). Conversely, of the 133 cases with 1p-, only 35 (26%) also showed dmin. Hence, the clustering of imbalances into whole-chromosome gains, losses, and structural changes by PCA was not always translatable into straightforward concurrence of abnormalities within cases belonging to the same cluster. Nevertheless, the PCA of genomic imbalances suggested that most NBs could be classified into groups based on the presence in the karyotype of whole-chromosome gains, whole-chromosome losses, or structural changes.

### Principal component analysis of cases

**Figure 2:**
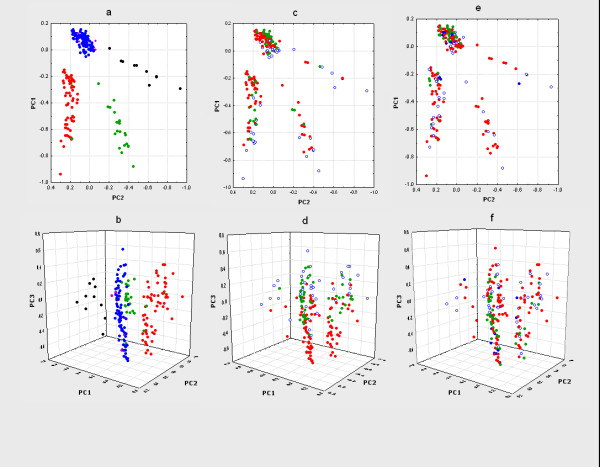
Clustering of tumour cases based on chromosomal imbalances using principal component analysis. In a, c, and e, two-dimensional representations based on the 1st and 2nd principal components are shown and in b, d, and f, three-dimensional representations are shown. In essence, the three-dimensional representations show a side-view and the two-dimensional representations a top-view of the clustering results. Colour codes in a and b: red, cases with 1p- without hsr/dmin; green, cases with 1p- and hsr/dmin; black, cases with dmin without 1p- ; purple, cases with hsr without 1p-; blue, other cases. Colour codes in c and d: red, tumours containing a higher number of whole-chromosome losses than whole-chromosome gains; green, tumours containing a higher number of gains than losses; empty circles, tumours not containing any whole-chromosome abnormalities. Colour codes in e and f: red, high risk tumours; green, low risk tumours; blue, intermediate risk tumours; empty circles, tumours with insufficient data for classification.

In order to further explore the subdivision of NB according to cytogenetic imbalances, we performed PCA in the reverse fashion, i.e. using the genomic imbalances as observations and the cases as variables. A scatter plot of all 259 cases according to the two first principal components resulted in four clusters (Figure 2a and 2b; Table 2): (1) those with dmin without 1p-, (2) those with 1p- as well as dmin or hsr, (3) those with 1p- without dmin or hsr, and (4) those with neither 1p-, nor dmin. Some structural imbalances other than dmin, hsr, and 1p- also showed an uneven distribution among the four groups, with +17q being particularly prevalent in group 3 and 11q- being more common in groups 3 and 4 than in groups 1 and 2. Cases with hsr without 1p- or dmin located separately. However, hsr was present only in 16 cases, of which 10 had either dmin, 1p-, or both. When adding the third principal component, groups 3 and 4 could be further sub-classified into karyotypes dominated by whole chromosomes gains and karyotypes dominated by whole-chromosome losses (Figure 2c and 2d). In group 4, whole chromosome gains were generally much more prevalent than in the other three groups, whereas group 3 was generally dominated by losses. Groups 1 and 2 showed only little variability along the third principal component, most probably because whole chromosome imbalances were rare in both these subgroups.

**Table 2:** Cytogenetic subgroups derived from PCA^1^

Chromosomal imbalances	1p- negative dmin positive	1p- positive dmin/hsr positive	1p- positive dmin/hsr negative	1p- negative dmin/hsr negative
Number of cases	21	40	93	97

*Whole chromosome gains*				
+1	0	8	3	9
+2	0	3	3	**10**
+3	5	0	2	**10**
+5	0	3	3	**12**
+6	0	0	6	**13**
+7	5	3	**11**	**25**
+8	0	3	5	**12**
+9	0	5	4	**16**
+11	**10**	0	3	**10**
+12	0	3	8	**20**
+13	5	3	5	**18**
+17	5	0	**10**	**32**
+18	5	3	**12**	**18**
+20	0	5	**10**	**15**
+21	5	3	2	**14**
*Whole chromosome losses*				
-2	0	8	4	**10**
-3	0	5	**16**	**16**
-4	0	5	**13**	**24**
-5	0	8	5	**12**
-6	5	5	9	**13**
-8	**10**	8	**10**	**12**
-9	0	8	**13**	**15**
-10	5	**13**	**15**	**19**
-11	0	8	**14**	**24**
-12	0	0	6	9
-13	0	3	**16**	**11**
-14	**10**	8	**13**	**12**
-15	0	**10**	**19**	**16**
-16	5	8	6	**19**
-17	5	**13**	**12**	**12**
-18	0	5	4	9
-19	**10**	5	**13**	**22**
-20	5	5	9	**12**
-21	5	8	**13**	**14**
-22	5	5	**13**	**15**
-X	**14**	5	**27**	**29**
*Partial chromosome gains*				
+1q (1p31q44)	**19**	**13**	**13**	**21**
+17q (17q12q25)	5	8	**20**	**12**
*Partial chromosome losses*				
1p- (1p36p11)	0	100	100	0
3p- (3p26p12)	**10**	5	6	**12**
6q- (6q23q27)	5	5	9	6
11q- (11q23q25)	5	3	**10**	**11**
*Gene amplifications*				
hsr	0	23	0	0
dmin	100	90	0	0

^1^Imbalances present at a frequency of 10% or more are in bold type; imbalances included in sub-group definitions are underlined.

### Correlation to age, stage, and risk classification

**Figure 3:**
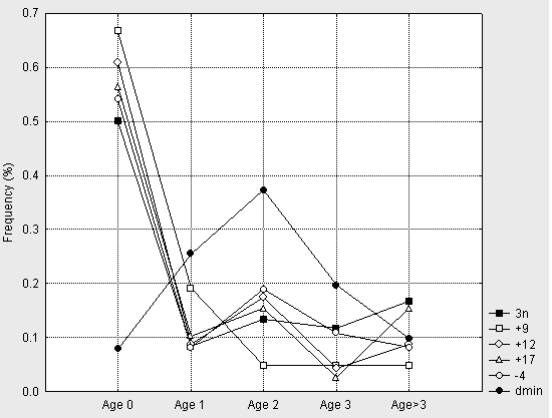
Frequencies across age groups. Lines represent triploid cases, cases with +9, +12, +17, -4, and dmin in patients aged 0 (below 1), 1, 2, 3, and above 3 years of age.

Age and tumour stage could be identified in 252 and 217 cases, respectively. In many of the original publications, age was given in full years only and more refined age sub-division could therefore not be made. Age and stage were combined into a simplified clinical risk classification as described in Materials and Methods. This resulted in the identification of 48 low-risk, 24 intermediate-risk, and 146 high-risk patients. To establish whether any of the selected imbalances were present more or less often than expected from a random distribution we first calculated the expected number of tumours with a certain imbalance for each sub-group and compared these distributions to the true distributions in our material. Suspected over/under-representations were tested with Chi-square test. With respect to the age of tumour presentation, tumours with a near-triploid karyotype as well as tumours with gains of whole chromosomes 9, 12 and 17 (i.e. abnormalities characteristic of group 4) were all over-represented in patients less than 1 year of age (Figure 3). The same abnormalities were over-represented in tumours of lower stages (1–2) and in low-risk tumours (Figure 2e and 2f). In addition, loss of whole chromosome 4 was over-represented in low-stage (1–2) and low-risk tumours. In contrast, 1p- and dmin (characteristic of groups 1–3) were over-represented in high-risk tumours; dmin were also over-represented in patients >1 year and <4 years of age.

### Temporal development of chromosome changes

**Figure 4:**
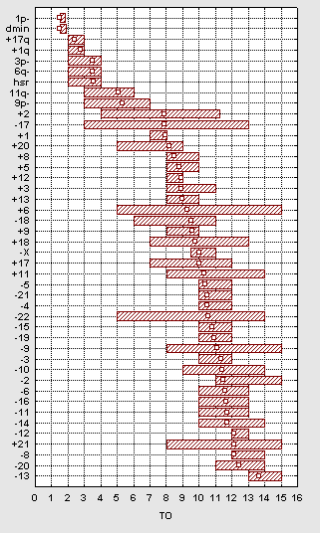
Temporal analysis. The estimated TO values are indicated by open squares. The range of the 25th and 75th quartiles is indicated by filled boxes. The imbalances are ordered with respect to their estimated TO values.

The total number of imbalances in a tumour can be used as an approximation of its state of clonal evolution [8-12,18]. Hence, the modal number of imbalances at which a specific cytogenetic abnormality occurs can be used to approximate its time of occurrence (TO) in relation to other abnormalities in the same sample of tumours. The present TO analysis showed that the structural rearrangements appeared in a relatively specific order, initiated by 1p- and dmin (TO = 1–2), followed by +17q, +1q, 3p-, 6q-, hsr (TO = 2–4), and finally by 11q- (TO = 3–6) (Figure 4). For the whole-chromosome changes, the TO values were confined between 8 and 14, with highly overlapping 25th–75th percentile intervals. Accordingly, there was no difference in TO between gains and losses of whole chromosomes. Hence, each whole-chromosome abnormality was most probably acquired together with one or several other abnormalities of the same type.

## Discussion

Studies of the tumour genome by chromosome banding analysis suffer from several shortcomings, e.g. difficulties in obtaining a sufficient number of mitotic cells for analysis, overgrowth of normal cells in tissue culture, poor quality of chromosome preparation, and an inherently low resolution. Nevertheless, several large cytogenetic series of NB, some of which have been complemented by multicolour fluorescence in situ hybridisation analysis, have been reported with a high frequency of cases showing cytogenetic abnormalities and with detailed karyotype descriptions [6,21-25]. In the present study, we have summarised the published cytogenetic data in NB and performed a comprehensive and unbiased statistical analyses. PCA of chromosomal imbalances revealed three distinct clusters of imbalances that tended to occur together in the same tumours, i.e. whole-chromosome gains, whole-chromosome losses, and structural changes. Of the structural changes, 1p- and dmin were by far the most common, occurring in 48% and 20% of the tumours, respectively. Indeed, when PCA of the tumour cases was performed, these two changes largely dominated the first two principal components. The third principal component was dominated by the spectrum of whole chromosome changes. In summary, the PCAs indicated that the accumulated cytogenetic data could warrant sub-classification of NBs into at least four groups, i.e. those characterised by (1) dmin and few other imbalances, (2) dmin, 1p-, and few other imbalances, (3) 1p-, other structural changes such as +17q, and whole chromosome losses, and finally (4) whole-chromosome gains and losses. Groups 1–3 were dominated by the structural abnormalities associated with high-risk tumours in the present study (dmin/hsr, 1p), while group 4 was dominated by numerical abnormalities associated with low-risk tumours (+9, +12, +17, and -4).

Several authors have suggested that NB can be sub-divided into at least two biological entities, reflecting the heterogeneous scenario observed in NB patients [26]. The first group is characterised by a near-diploid karyotype and genetic imbalances based on structural cytogenetic changes. Among these, the NB with *MYCN *amplification, also often showing 1p-, generally present with high-stage disease and are rapidly progressive, whereas the tumours without *MYCN *amplification but with other structural changes such as loss of heterozygosity for 11q typically occur in patients with slowly progressive but often fatal disease. These two subgroups most closely resemble groups 1–2 and 3, respectively in the present study. The other main biological group is characterised by a near-triploid karyotype with few structural chromosome changes, occurring in infants with confined disease and a very good prognosis. This group corresponds to our group 4. Hence, despite its inherent methodological limitations, the present study identified clinico-genetic sub-groups that were largely similar to those identified by other recent studies using comparative genome hybridisation and oligonucleotide arrays [3,15].

It has been suggested that diploid and triploid NB may arise from a common tetraploid precursor undergoing tripolar cell division [26,27]. The first type of daughter cell (2n) may give rise to the near-diploid, typically aggressive, tumours characterised by structural chromosomal changes, particularly 1p-, 11q-, +17q, and dmin (our groups 1–3). The second type of daughter cell (3n) may evolve into the near-triploid, typically indolent, tumours harbouring whole-chromosome changes, such as +9, +12, and +17 (our group 4). Although a highly attractive hypothesis, little empirical data have so far been presented to substantiate that these two biological NB entities in fact evolve through different evolutionary pathways. If the near-diploid tumours were actually derived from a tripolar tetraploid mitosis, segregating into haploid sets as originally suggested by Kaneko and Knudson [27], this could result in three possible sex chromosome complements in the resulting 2n cells, i.e. XY, XX, and YY. Of these, XY would be twice as probable as each of the two other two. It can be further argued that the YY cells, having nullisomy for the X chromosome, are not likely to survive. Hence, if the 2n tumours resulted from a tripolar cell division of a tetraploid cell, this would imply that 1/3 of the NB with 2n ploidy in male patients should have an XX chromosome complement. None of the 90 karyotypes from male patients with 2n tumours in the present series, showed such sex-reversal. However, this chain of reasoning assumes that somatic cell feminisation would not be lethal on the cellular level, which cannot be excluded at present. Furthermore, it does not rule out that tripolar division of a tetraploid cell lies behind the generation of near-triploid tumours, in which such somatic sex reversal would not occur if one assumed segregation into haploid sets.

In the present study we used the number of chromosome imbalances at which a certain chromosomal aberration typically occurred to model the evolution of genomic changes in NB. This approached has previously been used to delineate the temporal development of chromosome aberrations in several other tumours [9-11,28,29]. Our analysis showed that the structural changes occurred in a step-wise fashion, with loss of 1p sequences and the emergence of double minute chromosomes as the earliest cytogenetic events, followed by gain of 1q and 17q, loss of 3p and 6q, acquisition of homogeneously staining regions, and finally loss of 11q. In contrast, the gains and losses of whole chromosomes occurred more or less simultaneously, in a fashion similar to that suggested for the acquisition of whole-chromosome gains in hyperdiploid childhood leukemias [30]. This supports the notion that the numerical chromosome changes in 3n NB may arise through a single rare event, such as an asymmetrical mitotic cell division. In contrast, the 2n tumours appear to develop through the sequential acquisition of structural changes. Such a step-wise evolution of genome imbalances has been found in many common carcinomas, such as colorectal cancer, breast cancer, ovarian cancer, and bladder cancer [8]. It is possible that this step-wise acquisition of clonal changes reflects genome plasticity, which might explain why 2n tumours are more resistant to treatment than 3n tumours.

## Conclusion

Our finding of different dynamics for the acquisition of chromosome changes in the two main biological subsets of NB, taken together with the PCA classification of tumours into those with structural and those with numerical changes, lends strong support to the hypothesis that high-risk and low-risk NBs are biologically diverse entities, evolving through at least two distinct genetic mechanisms.

## Abbreviations

2n – near-diploid; 3n – near-triploid; dmin – double minute chromosomes; hsr – homogeneously staining regions; NIPT – number of imbalances per tumour; NB – neuroblastoma; TO – time of occurrence.

## Competing interests

The author(s) declare that they have no competing interests.

## Authors' contributions

DG and MH collected cytogenetic data, performed statistical analyses and drafted the manuscript. GL and IØ collected clinical data and drafted the manuscript. All authors have read and approved the final manuscript.
